# Assessment of Intraepithelial Lymphocytes Count in Potential Celiac Disease

**DOI:** 10.1111/apm.70015

**Published:** 2025-03-18

**Authors:** Roberta Mandile, Mariantonia Maglio, Antonella Marano, Luciano Rapacciuolo, Valentina Discepolo, Riccardo Troncone, Renata Auricchio

**Affiliations:** ^1^ European Laboratory for the Investigation of Food Induced Diseases (ELFID) University of Naples Federico II Naples Italy; ^2^ Department of Translational Medical Sciences, Section of Paediatrics University of Naples Federico II Naples Italy

**Keywords:** histology, IELs, immunohistochemistry, Marsh 0, Marsh 1

## Abstract

Intraepithelial lymphocytes (IELs) count, central for coeliac disease (CD) diagnosis, can be performed either directly on hematoxylin and eosin (H&E)–stained paraffined sections or on optimal‐cutting‐temperature‐compound (OCT)‐embedded frozen sections stained by immunohistochemistry (IHC) with anti‐CD3. We evaluated the concordance in Marsh grading between these two techniques on a large sample of sections. A total of 280 patients with a normal intestinal architecture, 210 potential celiac disease (PCD) patients, and 70 controls (CTR) were included. At the H&E histological evaluation, 136/280 were classified as Marsh‐0 (showing < 25 IELs/100 enterocytes) and 144 Marsh‐1, while at the IHC evaluation, 191 were classified as Marsh‐0 (showing ≤ 34 CD3+/mm of epithelium) and 89 Marsh‐1. The overall concordance was 66.8% (48.6% Marsh‐1 and 86% Marsh‐0) with a Cohen Kappa value of 0.33. In the PCD group, the overall concordance was 63% (45.6% Marsh‐1 and 84% Marsh‐0) with a Cohen Kappa value of 0.26, while in the CTR group it was 77% (60% Marsh‐1, 90% Marsh‐0) with a Cohen Kappa value of 0.54. Differences between the two groups were statistically significant (*p* < 0.05). In conclusion, the concordance of IELs counts between histological and IHC evaluation is low (Kappa Cohen 0.54) in no‐CD and even more in PCD patients (0.26). Caution must be paid when classifying a patient as Marsh‐0 or Marsh‐1 according to the technique used.

## 1 Introduction

Intraepithelial lymphocytes (IELs) represent a normal component of the small intestinal mucosa [1]. Increased density of IELs is reported in different disorders, including infections, small bowel bacterial overgrowth, tropical sprue, autoimmune disorders, and drug reactions [2]. However, their marked increase in the intestinal epithelium, especially when associated with circulating anti‐tissue transglutaminase2 (anti‐TG2) and/or anti‐endomysium antibodies (EMA), is a key marker of celiac disease (CD) [3]. In this condition, inflammatory cytokines released by gluten‐specific CD4+ T cells activated in lamina propria contribute “to arm” the increased IELs to kill epithelial cells expressing stress markers, leading to the progressive development of enteropathy [4]. Nevertheless, an increased number of IELs can also be dissociated from villous atrophy (VA). Histological evaluation remains the gold standard for CD diagnosis. It must be necessarily performed in the case of anti‐TG2 positivity, regardless of the presence of symptoms, when anti‐TG2 levels are lower than 10 times the upper limit of normality of the cutoff. When anti‐TG2 are positive at a very high level (above the 10 times the upper limit of normality), the biopsy can be omitted since there is an almost 100% probability of finding an atrophic mucosa [5]. The intestinal damage in CD can range from minor or even absent histological disruption to a completely destroyed architecture. Indeed, the term potential celiac disease (PCD), first adopted by Ferguson and colleagues [6], refers to a subset of patients that produce CD‐specific autoantibodies but present a normal intestinal architecture. The duodenal histology of these patients can be completely normal or display an increase in IELs density, respectively Marsh 0 (M0) or Marsh 1 (M1) lesion according to historical Marsh‐Oberhuber classification [7]. In recent years, however, the use of qualitative classifications for the description of mucosal lesions in CD, like the Marsh‐Oberhuber classification, has been discouraged due to the scarce intra‐and inter‐observer reproducibility and has been replaced by quantitative histology [8], where the mucosal damage is measured and numerically expressed. Villi length and crypt depth must be counted: the villi to crypts ratio is defined as normal when it is higher than 2 [9]. Equally, IELs should not be just globally evaluated as “normal” or “increased” but their precise number must be reported in a correct histological assessment. IELs count is of primary importance, especially in the context of a PCD diagnosis, where M1 and M0 patients have different chances to develop a VA over time if maintained on a gluten‐containing diet.

The presence of IELs is usually evaluated on hematoxylin and eosin (H&E) stained duodenal sections; however, because of the scarce contrast with the epithelial cells, the presence of intra‐epithelial lymphocytosis may not always be easily recognizable. A more recent alternative for IELs count is the targeting of IELs surface cell marker CD3 by immunohistochemistry (IHC) performed on optimal cutting temperature compound (OCT)‐embedded frozen sections. Thanks to the precise targeting of CD3+ cells, IHC should theoretically be more precise than H&E histological evaluation. Since diagnostic criteria based on H&E counts may not be applicable to CD3‐based IHC counting, we aimed to evaluate the concordance between these two techniques on a large sample of sections.

## 2 Patients and Methods

### 2.1 Biopsies

We retrospectively collected data from 280 patients diagnosed by small intestinal histology in our center between 2014 and 2021: all of them presented a normal intestinal architecture on a gluten‐containing diet. Clinical data were retrieved from their medical records. A total of 210 patients had high serum levels of anti‐TG2 antibodies (> 16 U/mL, laboratory cut‐off) and positive EMA confirmed on two consecutive blood samples and were thus classified as PCD patients, 70 had a negative CD‐associated serology and were labeled as controls (CTR).

### 2.2 Histology, Morphometry, and Immunohistochemistry

**FIGURE 1:**
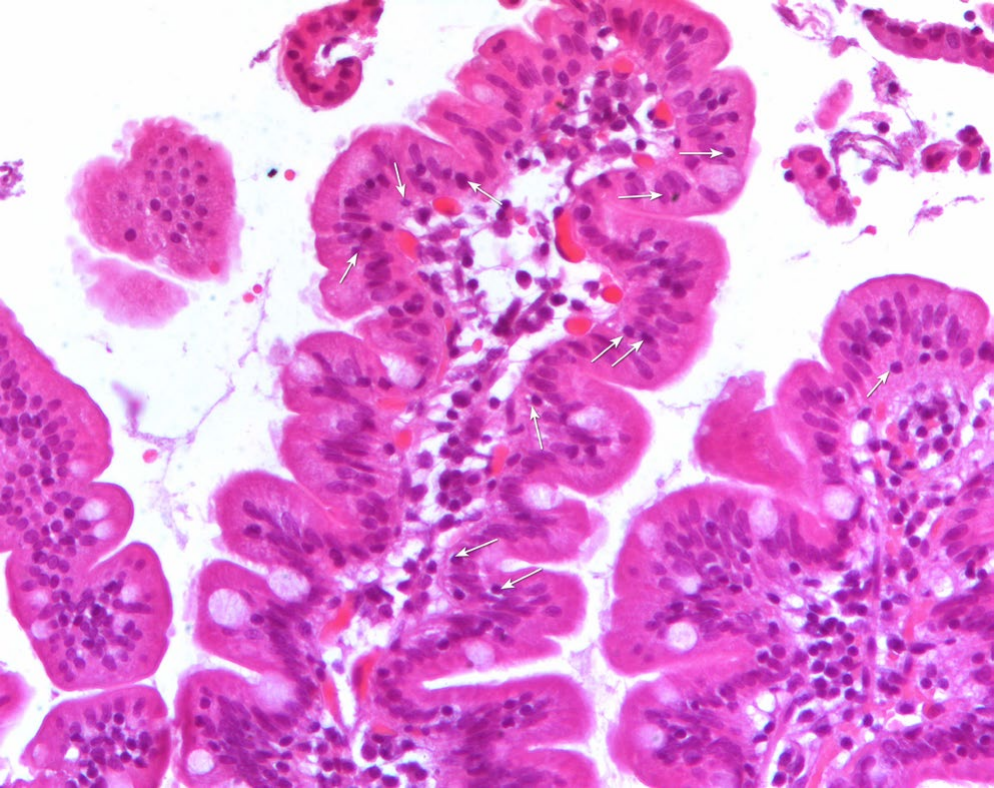
H&E‐stained paraffin‐embedded section of a patient with normal intestinal architecture showing high density of IELs (white arrows). 400× Magnification.

During esophagogastroduodenoscopy (EGDS) multiple duodenal biopsies were taken from the duodenum in each patient. At least three fragments were fixed in 10% formalin and embedded in paraffin. 4 μm thick sections, stained with H&E, according to routine protocol, were observed by a light microscope (Nikon Eclipse 80i) equipped with the software NiS‐Elements D 3.2 that allows histological and morphometrical analyses. The microscope is also equipped with the acquisition system Nikon DS‐Ri1. At least five sections per fragment had been analyzed by two expert operators in a blinded manner to any serology results. Villous height (Vh), crypt depth (Cd) and density of IELs were calculated on at least three well‐oriented villous/crypt units, and 300 enterocytes per fragment were evaluated. Vh/Cd ratio > 2 was considered normal [9]. IELs, appearing as small cells with an intense hematoxylin‐stained nucleus, were directly counted (Figure 1). Among biopsies with a normal Vh/Cd, those showing less than 25 IELs per 100 enterocytes were coded as M0, while those with a number of IELs ≥ 25 per 100 enterocytes were coded as M1, according to the Marsh‐Oberhubehr classification [7]. The Marsh score was assigned based on the score of the fragment with the worst picture.

**FIGURE 2:**
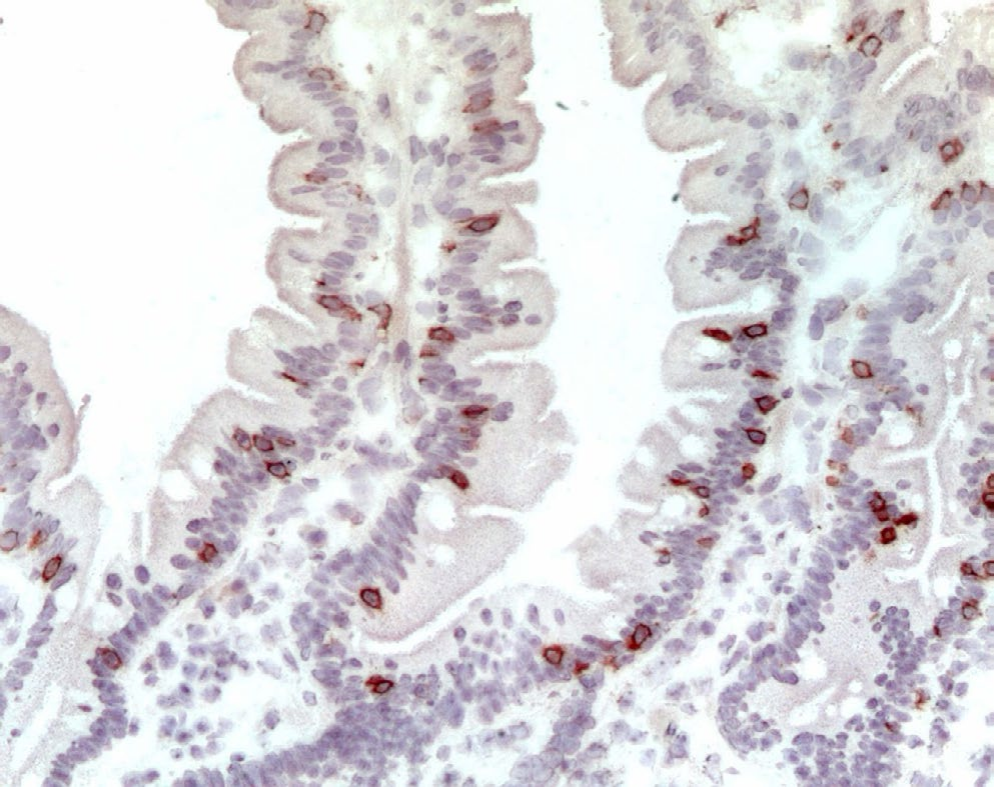
CD3^+^ IELs (brown cells) stained by IHC on OCT embedded frozen section of a patient with normal intestinal architecture. 400× Magnification.

The last duodenal fragment available was embedded in an optimal cutting temperature compound (OCT) (Killik; BioOptica, Milan, Italy), stored in liquid nitrogen, and used for immunohistochemical staining for CD3+ (Figure 2), previously reported [10]. The number of stained cells per millimeter of epithelium determined the density of CD3+ cells in the epithelial compartment; the cut‐off value to distinguish between M0 and M1 patients was 34 cells per mm of epithelium [9]. Counting was performed by two expert operators with an Axioscop Zeiss microscope using a 40× objective and an ocular equipped with a ruler that is placed parallel to the basal membrane of the epithelium. At least 4 mm of epithelium per fragment was analyzed.

### 2.3 Statistics

GraphPad Prism Software (Version 6, GraphPad Software, San Diego, CA, USA) was used to perform statistical analysis. We recurred to an unpaired *t*‐test to compare continuous variables between the groups. The *χ* ^2^ served to compare percentages. We applied the Bland–Altman method and calculation of the coefficient of variability to evaluate intraobserver and interobserver variations in IELs count on H&E stained sections and CD3+ count on IHC stained ones. The concordance between the two techniques in the definition of the Marsh grade was obtained by the Cohen K calculation.

## 3 Results

### 3.1 Concordance Between Histological and Immunohistochemical Evaluation in Marsh Grade Definition

Among CTR patients, clinical reasons that led to an intestinal biopsy were epigastric pain in 26/70 (37.1%) patients, suspicion of inflammatory bowel disease (IBD) in 8/70 (11.4%), dysphagia in 6/70 (8.6%), failure to thrive in 3/70 (4.2%), and other clinical indications in 27/70 (38.6%). Mean age at diagnosis was comparable between PCD and CTR groups (8.6 years vs. 10.3 years, *p* = 0.14) as well as the female to male ratio (70% vs. 65%, *p* = 0.2). IgA deficiency was excluded.

**FIGURE 3:**
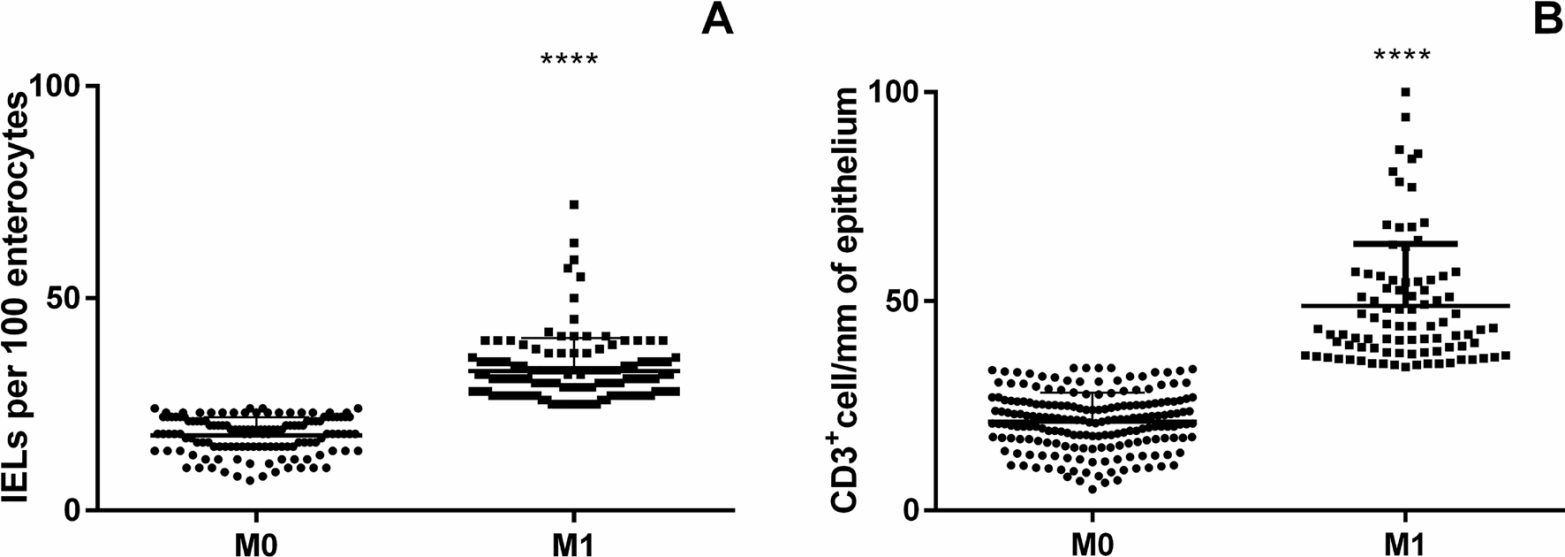
Intraepithelial lymphocytes (IELs) count. (A) Density of IELs per 100 enterocytes at the histological evaluation on H&E sections. (B) Density of CD3+ cells per mm of epithelium at the immunohistochemical evaluation on OCT sections. M0: Marsh 0, M1: Marsh 1. Unpaired *t*‐test, ****p < 0.0001.

At the H&E histological and morphometrical evaluation, all patients had a villi/crypts ratio > 2. Based on IELs count (cut‐off 25 IELs/100 enterocytes), 136 patients were classified as M0 and 144 as M1. The mean of IELs per 100 enterocytes was 17.7 (95% CI = 16.95–18.45) for M0 and 32.8 (95% CI = 31.5–34.3) for M1 patients (Figure 3A) (*p* < 0.001).

**FIGURE 4:**
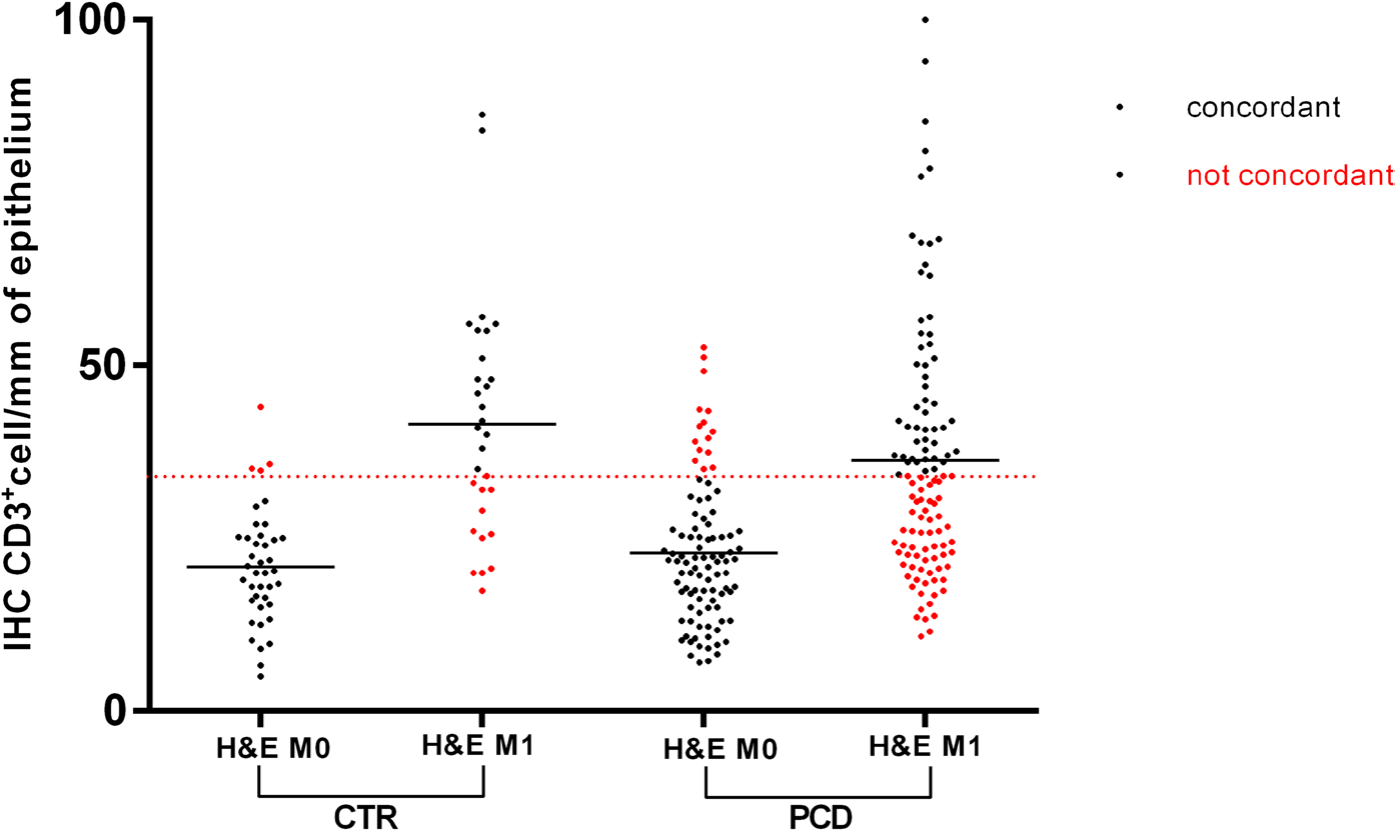
CD3^+^ IELs in CTR and PCD with M0 and M1 histology. CTR: Controls; PCD: Potential celiac disease; H&E M0: Histology Marsh 0; H&E M1: Histology Marsh1. Red line represents the cut‐off value for CD3+ cells, it is of 34 positive cells/mm of epithelium.

**FIGURE 5:**
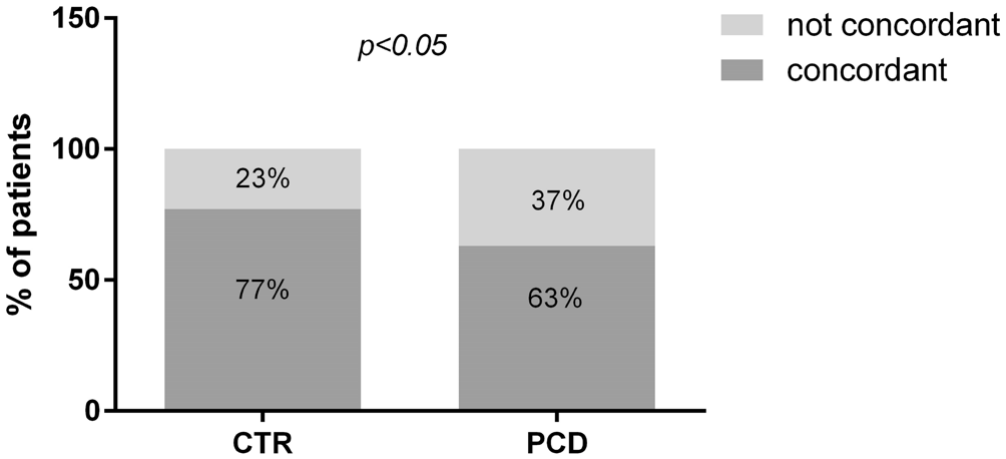
Concordance of IELs count between histology and immunohistochemical staining in CTR and PCD subjects. CTR: Controls; PCD: Potential celiac disease. The *χ* ^2^ test was applied.

At the IHC evaluation with CD3+ targeting (cut‐off 34 positive cells/mm of epithelium) 191 out of 280 patients were classified as M0 and 89 as M1. Mean density of CD3+ cells was 21.4 per mm of epithelium (95% CI = 20.25–22.22) for M0 and 48.84 (95% CI = 45.7–51.9) for M1, *p* < 0.0001 (Figure 3B). The overall concordance between the two methodologies of IELs count was 66.8% (187/280 patients) ranging from 48.6% for M1 to 86% for M0. We performed the Cohen Kappa analysis to evaluate the degree of concordance. The analysis showed a value of 0.33 (Table 1). We performed the same analysis considering separately PCD and CTR patients (Figure 4). In the PCD group, the overall concordance was 63% (133/218 patients), ranging from 45.6% for M1 to 84% for M0. The Cohen Kappa value was 0.26. In the CTR group, the overall concordance was 77% (54/70 patients), ranging from 60% for Marsh 1 to 90% for Marsh 0. The Cohen Kappa value was 0.54 (Table 1). Differences observed between the CTR and PCD groups were statistically significant (*p* < 0.05 chi‐squared test) (Figure 5).

**TABLE 1:** Summary table of concordance studies.

	Histological evaluation	CD3 ≤ 34/mm epithelium	CD3 > 34cells/mm epithelium	Relative concordance	Overall concordance	Cohen *K*
CTR + PCD (280 patients)						
M0	136	117	19	86%	66.8%	0.33
M1	144	74	70	48.6%		
CTR (70 patients)						
M0	40	36	4	90%	77%	0.54
M1	30	12	18	60%		
PCD (210 patients)						
M0	96	81	15	84%		
M1	114	62	52	45.6%	63%	0.26

Abbreviations: CTR, controls; M0, Marsh0; M1, Marsh1; PCD, potential celiac disease.

We performed on a subset of 20 patients the evaluation of intra‐ and interobserver variabilities in IELs count on H&E stained sections and CD3+ IELs count on IHC stained ones, assessed by Bland–Altman analysis. Our data showed that the mean differences in two series of evaluations were very low. These data were confirmed also by the assessment of the coefficient of intra‐and inter‐variability for both methodologies (Table 2). Finally, we evaluated the concordance in establishing the Marsh grade: intra‐and interobserver evaluations gave a concordance of 100% for the evaluation of H&E stained sections and of 95% for IHC stained ones.

**TABLE 2:** Bland–Altman analysis data. In the table, all parameters of intra‐and interobserver variation in counting of IELs on paraffin (above) and OCT sections (below), performed by the Bland–Altman test, are reported.

	Mean difference	Standard deviation	Limits of agreement	Coefficient of variability	Concordance of Marsh classification
IELs count/100 enterocytes (H&E)					
Intraobserver	0.75 (−10 to 10)	3.78	−6.66 to 8.16	10.7%	100%
Interobserver	0.85 (−4 to 10)	3.63	−6.26 to 7.96	12.3%	100%
CD3+ IELs count/mm of epithelium					
Intraobserver	1.74 (−3.75 to 11)	3.75	−5.61 to 9.10	12.4%	95%
Interobserver	1.87 (0 to 4)	1.67	−1.40 to 5.15	8.5%	95%

## 4 Discussion

Despite the latest ESPGHAN guidelines having widened the criteria to access the biopsy‐sparing approach, histological evaluation remains the gold standard for CD diagnosis and is mandatory for children with low levels of anti‐TG2 antibodies (< 10 times the upper limit of normality of the laboratory cut‐off) [8]. In this case, histological duodenal assessment can provide two different results: an overt duodenal damage, with a villi/crypts ratio < 2, or a preserved intestinal architecture, with a villi/crypts ratio > 2. In the first case, patients must be addressed to a gluten‐free diet regardless of the presence of symptoms. Vice versa, when a normal villi/crypts ratio is observed, patients receive a diagnosis of PCD and can be monitored on a gluten‐containing diet unless the presence of invalidating symptoms. This is supported by the fact that, even after a long‐term follow‐up, chronic gluten consumption does not seem to alter their growth and nutritional parameters [11] and only around 30%–40% of them will progress to full‐blown disease over time [12]. Among risk factors associated with future development of VA, it has been demonstrated that patients with a Marsh 1 lesion have more chances to develop full‐blown disease over time compared to patients with a Marsh 0 lesion. For this reason, the precise allocation into a Marsh group (M0 vs. M1) by IELs count is of primary importance for the clinical management of PCD patients.

Typically, IELs have been directly counted in paraffin‐embedded H&E‐stained sections, together with the morphological evaluation of the intestinal mucosa.

With this technique, the number of IELs is calculated in comparison to the number of epithelial cells and expressed as the number of IELs/100 enterocytes. Cut‐off values of normality have changed over time, starting from > 40/100 enterocytes and progressively reducing. In a recent large multicentric study designed by Rostami et al. [13], based on IELs counts on > 400 mucosal biopsy specimens, the cut‐off of 25 IEL/100 enterocytes emerged as the best to discriminate between M0 and M1 lesions. In recent years, together with the historical histological evaluation for IELs count, IHC analysis has become common to better identify different types of inflammatory cells both in the epithelium and in the lamina propria. Widely used in scientific studies, IHC has been recently recommended by the latest guidelines also in clinical settings in cases of doubtful or difficult CD diagnosis [9]. Despite there being no evidence for a supremacy of one of the two techniques for IELs count, most studies comparing the results of H&E with CD3 IHC have shown IHC to be more precise since it directly targets the T cell surface marker CD3+ [14, 15, 16, 17]. The only study conducted in PCD children by Pellegrino et al. on a small group of patients and controls showed no difference between the two techniques with comparable sensitivities and specificities [18].

In the present work, we aimed at investigating the concordance between the histological and immunohistochemical evaluation for allocation of patients in Marsh categories (M0 vs. M1), focusing on children with a PCD diagnosis. Our data confirm that the two techniques do not provide overlapping results as the rate of concordance is very low in patients with a normal intestinal architecture. This is particularly true for PCD patients, where the Kappa Cohen reaches the lowest value of 0.26. More in detail, our data show that histologic evaluation on paraffin sections tends to identify more IELs compared to IHC and, therefore, overestimates M1 lesions compared to IHC analysis on frozen sections. To confirm that this low concordance was really imputable to a difference between the two techniques (H&E and IHC) and not to observer variability, we performed on a subset of patients intra‐and inter‐observer evaluations: in both cases, the reproducibility was very high. Different hypotheses may be advocated to explain the scarce concordance between the two techniques. First, the two analyses were performed on different biopsy samples from the same patient, included respectively in paraffin and in OCT. Since patchy lesions are reported in CD [19], the scarce concordance could be related to a really different degree of mucosal infiltration. This limitation could be overcome by the use of IHC on paraffin sections, which has not been performed in our study. The use of IHC on paraffin sections presents several advantages: paraffin specimens are cheaper, easier to stock, and more importantly, morphometric and immunohistochemical evaluations can be performed on the same sample, overcoming the problem of patchy lesions. However, currently, only CD3+ cells and TCRγδ+ cells can be identified on paraffin sections, as antibodies anti‐CD25+, an IL2 receptor component, are not available. This consistently limits the evaluation of the intestinal mucosa status, as the lamina propria compartment with inflammatory mononuclear CD25+ cells cannot be studied. In contrast, IHC on OCT frozen sections allows a more complete analysis of the intestinal mucosa status with the identification, together with CD3 positive and TCRγδ+ positive cells, of CD25+. On frozen sections, also anti‐TG2(8 deposits in the intestinal mucosa can be identified through the double immunofluorescence technique. Their presence has revealed to be important in the prediction of future evolution to VA in different studies [20]. Moreover, while IELs count on H&E is expressed as the number of IELs/100 enterocytes and a cut‐off of 25 IELs per 100 enterocytes is universally accepted to distinguish M0 from M1 lesions [13], with IHC on frozen sections, IELs can be expressed both as the number of cells/100 enterocytes or number of cells/mm of epithelium, impairing the standardization across different studies [10]. The advantage of counting IELs on mm of epithelium relies on the fact that, especially in a damaged intestinal mucosa, the epithelium can be pseudopluristratified and the enterocytes become hard to be recognized and counted. For this reason, many centers (including our) prefer to express IELs on mm of epithelium [10, 21, 22, 23]. However, in both cases, no multicenter study has been realized to identify universally accepted cut‐offs of normality.

In conclusion, the concordance of IELs count between histological and IHC evaluation on OCT embedded frozen sections is low in the definition of Marsh grade, especially when considering PCD patients: histological assessment tends to overestimate M1 lesions compared to IHC assessment on frozen sections. Moreover, while the cut‐off value of normality for IELs count on H&E sections has been more accurately evaluated in recent years, the cut‐off of normality for IELs count on OCT sections has not been defined in large multicentric studies. Since correct classification is of primary importance in PCD patients to define their clinical management, caution must be paid when classifying a patient as M0 or M1 according to the technique used.

## Ethics Statement

The study was conducted in accordance with the Declaration of Helsinki and approved by the Ethics Committee of the University Federico II of Naples (protocol number: 173/2023, 9/24, 12 June 2024).

## Consent

Informed consent was obtained from all subjects involved in the study.

## Conflicts of Interest

The authors declare no conflicts of interest.

## Data Availability

The data that support the findings of this study are available on request from the corresponding author. The data are not publicly available due to privacy or ethical restrictions.
